# Lack of Evidence for Chloroquine-Resistant *Plasmodium falciparum* Malaria, Leogane, Haiti

**DOI:** 10.3201/eid1809.120605

**Published:** 2012-09

**Authors:** Ami Neuberger, Kathleen Zhong, Kevin C Kain, Eli Schwartz

**Affiliations:** Rambam Medical Center, Haifa, Israel (A. Neuberger);; University of Toronto, Toronto, Ontario, Canada (K. Zhong, K.C Kain);; Sheba Medical Center, Tel Hashomer, Israel (E. Schwartz);; and Tel Aviv University, Tel Aviv, Israel (E. Schwartz)

**Keywords:** malaria, chloroquine, resistance, susceptibility, Plasmodium falciparum, parasite, Haiti, vector-borne infections

## Abstract

*Plasmodium falciparum* malaria in Haiti is considered chloroquine susceptible, although resistance transporter alleles associated with chloroquine resistance were recently detected. Among 49 patients with falciparum malaria, we found neither parasites carrying haplotypes associated with chloroquine resistance nor instances of chloroquine treatment failure. Continued vigilance to detect emergence of chloroquine resistance is needed.

*Plasmodium falciparum* malaria, which has been eliminated from most of the Caribbean islands, is endemic to the island of Hispaniola (*1**–**3*) and is especially prevalent in Haiti. Epidemiologic data from Haiti are scarce, but malaria is probably endemic to most areas situated at altitudes <300 m. The town of Leogane, situated in Ouest Province 30 km west of Port-au-Prince, is no exception, and we have reported a high incidence of malaria among febrile patients during the peak malaria transmission season of November 2010–March 2011 (*1*).

In sharp contrast to the situation in most other malarial regions in the world, *P. falciparum* parasites in Haiti remain susceptible to chloroquine (*4**,**5*). To the best of our knowledge, clinical failure of chloroquine for prophylaxis or treatment of *P. falciparum* malaria has not been reported, and the Centers for Disease Control and Prevention recommends chloroquine as a first-line option for the treatment and prophylaxis of malaria in Haiti (*6*).

All studies reporting universal chloroquine susceptibility of *P. falciparum* parasites in Haiti were performed before the 1990s (*4**,**5*). However, a recent report from the Artibonite Valley north of Port-au-Prince documented that the *P.* *falciparum* chloroquine resistance transporter (*pfcrt*) haplotypes were detected in 5 (6%) of 79 blood samples from Haitians with blood smears positive for *P. falciparum* parasites (*7*). Although this report did not include clinical data, did not document clinical chloroquine treatment failure, and reported the detection of haplotypes associated with chloroquine resistance in a minority of all tested samples, the emergence of chloroquine resistance would be of great concern. Because epidemiologic data from Haiti are scarce, at best, and because the malaria surveillance system operating in the country is inadequate, undetected cases of chloroquine-resistant malaria might exist. We aimed to search for chloroquine-resistant cases either clinically or by detecting *pfcrt* haplotypes in *P. falciparum* isolates in Leogane, Haiti.

## The Study

The study was conducted during November 2010–May 2011 in a primary health care clinic in Ouest Province of Haiti. The clinic is situated in Leogane, a town of ≈200,000 residents, 30 km west of Port-au-Prince. The clinic is staffed by volunteer nurses and doctors from Israel and Canada and by local hired staff from Haiti. The clinic has been operating on a daily basis since November 2010 (*8*).

Malaria was diagnosed by using a rapid diagnostic test (RDT; Paracheck Rapid Test, Orchid Biomedical Systems, Goa, India) to detect histidine-rich protein II in patients with histories of compatible febrile illness. All patients visiting the clinic with undifferentiated fever were tested by using an RDT, and malaria was not diagnosed or treated in these patients without this laboratory confirmation. Microscopy was not available. Blood from patients with malaria was collected on filter paper blots, which were sent to the laboratory at the University of Toronto (Toronto, ON, Canada). A *P. falciparum* DNA sample was extracted from blotted filter paper blood samples and eluted in 100 μL of water by using QIAGEN columns (QIAGEN, Chatsworth, CA, USA) according to the manufacturer’s instruction. A 10-µL aliquot of the DNA extract was used in real-time PCR, and results were confirmed by using a nested PCR and sequencing as described (*9**–**11*). Nested PCR was performed to amplify the *P. falciparum* *pfcrt* fragment around amino acid residue 72–76. DNA sequencing of the 134 base-pair PCR product was performed to determine the amino acid haplotype of residues 72–76 as described (*9**,**10*). The lysine to threonine mutation at residue 76 (K76T) in *pfcrt* was present in all chloroquine–resistance strains, and the amino acid haplotype of CVMNK was present in chloroquine-sensitive strains (Table 1, Table 2).

**Table 1:** Amino acid haplotypes of *pfcrt* K76T polymorphism, Haiti*

Chloroquine-resistant strains	Chloroquine-sensitive strains
CVIET	CVMNK
CVMNT	
CVMET	
SVMNT	

*pfcrt, *Plasmodium falciparum* chloroquine resistance transporter.

**Table 2:** PCR primer sequences for *cytochrome b* of *Plasmodium falciparum* gene fragment *pfcrt*, Haiti*

Primer sequence	Product size
5′-CCGTTAATAATAAATACACGCAG-3′ (sense)	537 bp
5′-CGGATGTTACAAAACTATAGTTACC-3′ (antisense)	134 bp
5′-TGTGCTCATGTGTTTAAACTT-3′ (sense)	
5′-CAAAACTATAGTTACCAATTTTG-3′ (antisense)	

*pfcrt, *Plasmodium falciparum* chloroquine resistance transporter.

Forty-nine patients with a febrile illness were positive by RDT for falciparum malaria. Their mean age (± SD) was 24.1 ± 14.4 years, and 28 were male. Forty-eight patients were Haitians with uncomplicated falciparum malaria; 1 was an expatriate with severe malaria. The patient with severe malaria was transferred to the United States for care.

All patients initially received chloroquine. One patient developed an allergic reaction to chloroquine and was subsequently treated with artemether–lumefantrine. No patients returned to the clinic after receiving chloroquine, even though they were encouraged to do so if fever persisted; all medical services were provided without charge. Attempts to contact patients by telephone 1 month after completion of treatment had limited success. Only 8 were contacted successfully; all were asymptomatic after treatment with chloroquine alone.

*P. falciparum* DNA was detected in all positive RDT samples. We did not observe any false-positive RDT results. Further evaluation was conducted for haplotypes associated with the chloroquine resistance gene. A total of 49 samples were amplified and sequenced. All falciparum isolates carried *pfcrt* haplotypes encoding the amino acid chloroquine-susceptible haplotype CVMNK (residues 72–76). The prevalence of chloroquine-sensitive strains was 100%.

## Conclusions

Haiti has traditionally been considered a unique region of endemic chloroquine-sensitive falciparum malaria. However this situation was challenged by a recent report of circulating *P. falciparum* parasites possessing haplotypes associated with chloroquine resistance in ≈6% of blood samples obtained by passive and active case detection (*7*).

We did not detect any cases of chloroquine treatment failure; in many cases, however, complete follow-up 1 month after treatment was not possible. Nor did we detect any falciparum isolates with *pfcrt* haplotypes associated with chloroquine resistance.

In addition, as previously reported, we did not detect any cases of malaria in expatriates who were using chloroquine prophylaxis (*1*). These observations strengthen the current opinion that *P.* *falciparum* remains chloroquine sensitive in Haiti.

Our conclusions are subject to several limitations. Our study was conducted in Leogane, situated 30 km west of Port-au-Prince. The haplotypes associated with chloroquine resistance were detected previously in the Artibonite Valley (*7*) ≈100 km north of Port-au-Prince; thus, chloroquine susceptibility might have varied by geographical region. In addition, cases of malaria caused by chloroquine-resistant strains of *P. falciparum* could have been missed because of the small size of our cohort. Another limitation of our study is the incomplete follow-up of most patients. Conditions in Haiti after the 2010 earthquake make adequate follow-up difficult. However, because medical services and medications were provided free of charge and because patients were instructed to return if fever persisted or recurred, we find it unlikely that cases of chloroquine treatment failure were missed.

The issue of possible chloroquine resistance has implications for Haitians and for the large number of volunteers and expatriates employed by the various aid organizations living in Haiti since the earthquake. Our results support the current recommendation of chloroquine as a first-line choice for treatment of falciparum malaria and for malaria chemoprophylaxis in Haiti. However, continued vigilance to detect the emergence of chloroquine resistance is warranted.
